# Effect of Heat Treatment and Hot Isostatic Pressing on the Corrosion Behavior of Ti_6_Al_4_ V Parts Produced by Electron Beam Melting Additive Manufacturing Technology

**DOI:** 10.1021/acsomega.4c04218

**Published:** 2024-06-28

**Authors:** Mutlu Karasoglu, Mustafa Özgür Öteyaka, Evren Yasa, Evren Tan, Melih Cemal Kuşhan

**Affiliations:** † Department of Mechanical Engineering, Faculty of Engineering, Eskişehir Technical University, Eskişehir 26555, Turkey; ‡ Eskisehir Vocational School, Department of Electronic and Automation, Eskişehir Osmangazi University, Eskişehir 26250, Turkey; § Department of Mechanical Engineering, Faculty of Engineering and Architecture, Eskişehir Osmangazi University, Eskişehir 26480, Turkey; ∥ Advanced Manufacturing Research Centre/University of Sheffield, Sheffield S60 5TZ, U.K.; ⊥ Mechanical Design Technologies Department, ASELSAN Inc, EKIM Laboratory, Ankara 06200, Turkey; # Department of Aeronautical Engineering, Faculty of Engineering and Architecture, Eskişehir Osmangazi University, Eskişehir 26040, Turkey

## Abstract

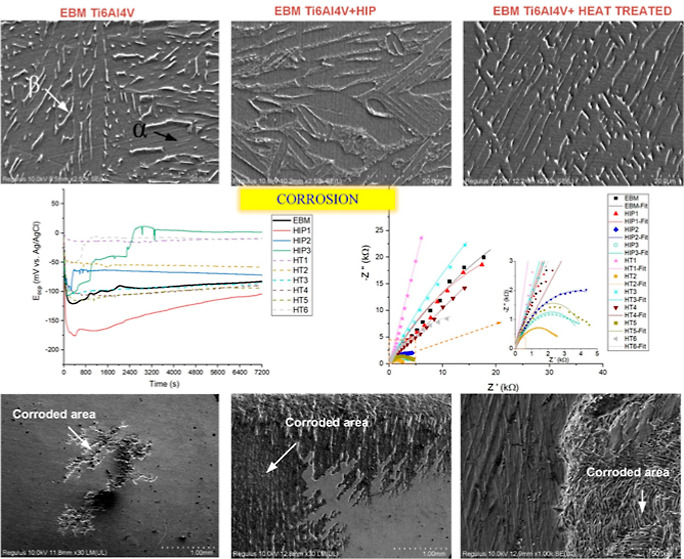

In this study, we investigated the effect of heat treatment (HT) and hot isostatic press (HIP) on the corrosion behavior of Ti_6_Al_4_ V, manufactured by electron beam melting (EBM) additive manufacturing. The preliminary results showed that the thermal process makes the columnar structure more pronounced and the α-lathe coarser compared to EBM. The β phase disappeared with the aging treatment and when increasing the HIP temperature treatment. According the open circuit potential (*E*_ocp_) behavior of samples, the HIP3 sample had performed more positive corrosion potential than rivals after 2 h of immersion probably due to equiaxed grain with coarser α-late and the absence of the β phase. In adverse, inferior corrosion behavior was observed for HIP1 because of a higher quantity of the β phase causing probably galvanic corrosion. The HIP process leads to a lower corrosion potential than EBM. At least one protective oxide layer formation was observed for all samples at the anodic branch, and the current density was lower for the HT3 sample. The microstructure analysis revealed the presence of the β-phase in the form of needle-like for the HT1 sample and HIP1 in the corroded area. Furthermore, the EDS line analysis showed the presence of aluminum with oxygen at the edge of the corrosion area for HIP1 suggesting aluminum plays a barrier against degradation. On the other hand, the HT1 showed higher impedance resistance due to the coarser α-lathe microstructure and well-defined β phase.

## 1 Introduction

Electron beam melting (EBM) is a powder bed fusion additive manufacturing (AM) technique commercialized and patented by the Swedish corporation ARCAM AB in 2001.^1^ The EBM process involves scanning and melting powder layers using a high-energy electron beam based on the Computer-Assisted Design model of the intended part. Parts are built up layer by layer. EBM serves many advantages including small beam spot size provides the ability to produce complex structures in comparison to large-scale AM processes like wire arc AM, lower residual stresses due to high preheating temperatures, and less need of support structures by virtue of slightly sintered powder layer. On the other hand, EBM has limitations, including restricted part size, extended processing times, and a limited choice of materials, primarily restricted to electrically conductive materials, as well as limited powder recyclability.^2^

Ti_6_Al_4_ V alloy is widely processed using EBM and is the most commonly utilized titanium alloy in AM. This is due to its outstanding properties, including high specific strength, a high melting temperature, superior fracture toughness, excellent biocompatibility, and high corrosion resistance.^3,4^ Ti_6_Al_4_ V is an α + β binary Ti alloy composed of both alpha stabilizing (Al) and beta stabilizing (V) elements.^5^ Ti_6_Al_4_ V alloys manufactured by the EBM process exhibit a distinctive microstructure that consists of columnar prior β grains aligned parallel to the build direction. Colony and lamellar α + β structures (Widmanstätten morphology) located within the prior β grains. Generally, the grain boundary α phase also exists along the borders of the prior β grains. The microstructure of the Ti_6_Al_4_ V alloy is a result of the thermal history encountered during the EBM process.^6^ The microstructural evolution can be summarized as follows: Initially, the melt pool solidifies into prior β grains. This is followed by rapid cooling from a temperature above the β transus (approximately 980 °C) down to the build temperature (600–650 °C), which transforms the prior β into α′ martensite.^7^ The beta transus temperature is the lowest equilibrium temperature at which the material transforms to 100% beta from alpha-plus-beta or alpha.^8^ Subsequently, near-isothermal annealing at the build temperature leads to the transformation of α′ martensite into binary α + β structures. Lastly, slow cooling from the build temperature to room temperature results in the decomposition of residual martensite at the top of the part.^7^ There have been numerous attempts to achieve equivalent grain structures in additive-manufactured Ti_6_Al_4_ V alloys using constitutional undercooling. However, it is challenging to achieve nucleation of equiaxed grains in the Ti_6_Al_4_ V alloy due to the narrow solidification range between the liquidus and solidus temperatures, which limits the degree of constitutional supercooling.^9^ Thermal variations in EBM result in heterogeneities in the microstructure, such as phase constitution transitions and changes in grain morphology and size.^10^ It was shown that α′ martensite is observed on the surface of Ti_6_Al_4_ V parts produced by the EBM technique.^6^ Some researchers have observed graded microstructures in EBM parts, and found that the microstructure becomes coarser with increasing build height. Additionally, an equiaxed to columnar transition of the primary β phase may occur at the lower side of the part due to the more conductive nature of the stainless-steel base plate, which leads to a higher degree of supercooling.^7^

EBM provides the capability to produce parts with a density exceeding 99% of the theoretical density.^11^ Yet, the presence of even a small number of defective pores within an EBM-produced component significantly impacts its mechanical properties particularly its fatigue performance.^12^ Moreover, anisotropic microstructure and defects lead to anisotropy in tensile test results.^13^ Microstructural heterogeneities also lead to local variations in the mechanical properties of the parts. It has been shown that graded microstructures influence the tensile and hardness test results. A decrease in strength and hardness was observed with increasing build height due to the coarser microstructure.^7^ Henceforth, thermal treatments and HIP processes are implemented to modify the microstructure and mitigate defects inherited from EBM process.^14,15^

It is well-established that the microstructure plays a crucial role in determining the mechanical properties of the Ti_6_Al_4_ V alloys. Numerous published works demonstrate that altering the microstructure through different heat treatments can lead to modifications in mechanical properties.^15−17^

Microstructure impacts not only the mechanical properties but also the corrosion behavior of the Ti_6_Al_4_ V alloys. Factors such as surface roughness, porosity, and microstructure, have significant influence on the corrosion of additively manufactured (AM) Ti_6_Al_4_ V and need improvement for long-term use.^18−22^ It has been shown that the corrosion resistance of Ti_6_Al_4_ V parts produced by EBM is enhanced due to the decreased surface roughness achieved through machining.^23^ Pores have the capacity to reduce the passivity of the alloy by augmenting the surface area in contact with the corrosive environment.^24^ Yeganeh et al. report that corrosion pits formed during corrosion testing of Ti_6_Al_4_ V alloy samples produced by EBM may be associated with gas pores.^25^ As previously noted, the microstructure formed post-EBM inherently exhibits anisotropy, a characteristic stemming from the nature of the process. Consequently, this can lead to directional discrepancies in corrosion behavior, similar to those observed in the mechanical properties. It has been shown by various researchers that the corrosion resistance of AM samples is greatly influenced by the build direction.^18,22,26−28^ For example, the effect of the XY and XZ-planes on the corrosion of Ti_6_Al_4_ V manufactured by EBM was investigated in 0.9 M NaCl solution.^22^ The microstructure of Ti_6_Al_4_ V manufactured by EBM exhibits fine Widmanstätten α + β structures. In this context, the XY-plane displays equiaxed grains, while the XZ-plane consists of larger β-grains along the construction direction. Report indicates that XY-planes, with their more homogeneous microstructure and higher grain boundary densities, demonstrate superior corrosion resistance compared to that of XZ-planes.

Some researchers have conducted comparative studies to evaluate the corrosion performance of conventional wrought and electron beam melted Ti_6_Al_4_ V components. According to Gai et al., the EBM-manufactured Ti_6_Al_4_ V exhibits superior passivation due to its smaller grain size and higher density of grain boundaries compared to the wrought alloy.^29^ It should be noted that the grain boundaries support oxide film nucleation and growth. Also, they found that the surface of EBM Ti_6_Al_4_ V was composed of a TiO_2_ and Al_2_O_3_ sublayer of oxides TiO_*x*_ and AlO_*y*_. In contrast, the wrought alloy had the lowest fraction of TiO_2_. Similarly, the EBM Ti_6_Al_4_ V, with a higher β phase and finer lamellar α/β phase, exhibited superior corrosion resistance compared to the wrought alloy in a phosphate-buffered saline solution. This was attributed to the reduced galvanic effect between the two phases (α and β) and the higher density of the oxide film.^30^ The influence of the β phase quantity on the corrosion resistance of EBM Ti_6_Al_4_ V remains uncertain and requires further clarification.

The pitting corrosion of EBM Ti_6_Al_4_ V depends on many factors such as grain size, grain boundaries, and percentage of phase distribution, roughness, and porosity.^21,22,31^ Generally, the Ti_6_Al_4_ V alloy is resistant to chloride but susceptible to bromide solutions. Abden and Palmer’s findings indicate that both EBM-produced and wrought alloy Ti_6_Al_4_ V samples display similar corrosion resistance, maintain passivity, and exhibit comparable pitting potential in a 3.5 wt % NaCl solution, irrespective of porosity.^18^ The corrosion performance of EBM Ti_6_Al_4_ V was also evaluated in a simulated body fluid. The results indicated that EBM Ti_6_Al_4_ V exhibited excellent corrosion resistance, particularly at potentials exceeding 1.5 V, and showed resistance against crevice corrosion.^32^

The HTs and HIP processes applied to improve the mechanical properties of Ti_6_Al_4_ V components produced by EBM may also influence their corrosion behavior as they affect the microstructure. However, the effect of thermal treatments on the corrosion behavior of the EBM Ti_6_Al_4_ V has not been extensively studied. According to the corrosion test results conducted by Carrozza et al. in a stimulated body fluid, they reported that HT at 680 °C for 4 h, followed by furnace cooling, did not have a significant effect on the corrosion behavior.^33^ Xiu et al.^34^ found that postprocessing of EBM Ti_6_Al_4_ V revealed that the β phase, rather than the α phase, exhibited better resistance to pitting corrosion. Furthermore, employing a HT involving solution annealing and air-cooling, followed by aging, resulted in improved corrosion resistance. Yeganeh et al., in their study applying a HT at 1000 °C for 1 h after EBM, investigated the effects of different cooling conditions (furnace cooling, air cooling, quenching). According to electrochemical analyses, they reported that the as-built samples exhibited the highest corrosion resistance in the early stages of corrosion tests, while after 1 month, all samples showed similar corrosion performance. The researchers attributed the superior corrosion performance of the as-built samples to a higher amount of β phase ratio.^25^

Another postprocessing investigated to increase the corrosion resistance of AM Ti_6_Al_4_ V is the hot isostatic pressing (HIP) process. The advantages of this technique include the improvement of mechanical properties through the reduction of residual stress and porosity in the AM parts, particularly enhancing fatigue properties and elongation.^35,36^ Additionally, the use of HIP for consolidating in situ shell structures produced by EBM is being researched as an emerging technology to enhance the productivity of the EBM process. In this method, parts are produced with a shell structure by forming only their outer shells while retaining a sintered powder at the core. The parts are then consolidated using HIP. This also provides microstructure control, enabling the production of parts with equiaxed or mixture of columnar and equiaxed grains, in addition to the typical columnar microstructure.^37^ Leon et al. conducted HIP treatment on EBM Ti_6_Al_4_ V by applying a pressure of 100 MPa at 925 °C for 3 h. Their analysis of the results suggests an improvement in the corrosion resistance of HIPed EBM Ti_6_Al_4_ V due to the growth of the β phase, along with a decrease in the α + β phase.^38^ Szymczyk-Ziółkowska et al. found that Ti_6_Al_4_ V postprocessed with HIP exhibited lower corrosion resistance when compared to the as-built EBM counterparts.^23^ They found that the corrosion potential of the HIP-treated sample was −0.15 V more cathodic compared with the as-built sample. In contrast, a stable and thick passive layer was observed upon analysis of the anodic branch of the polarization test.

As reported above, the literature provides limited insights into the effects of HT and HIP postprocesses parameters on the corrosion resistance of EBM Ti_6_Al_4_ V, emphasizing the necessity for a more comprehensive investigation. Therefore, in this study, a solution treatment was conducted, followed by aging processes at various temperatures and durations. Additionally, to explore the effects of the temperature and pressure, different temperature and pressure conditions were applied in the HIP process. After HT and the HIP process of EBM Ti_6_Al_4_ V, the microstructure modification was systematically analyzed and compared to as-built EBM specimens. Furthermore, the corrosion resistance of the as built and treated EBM Ti_6_Al_4_ V in 3.5 wt % NaCl solution was assessed using electrochemical techniques such as open circuit potential (OCP), potentiodynamic polarization, and electrochemical impedance spectroscopy (EIS).

## 2 Materials and Methods

### 2.1 Sample Fabrication and Heat Treatment

The samples used in this work were produced in a cubic geometry with dimensions of 25 mm × 25 mm × 25 mm by using the EBM technique with an ARCAM A2X machine. To achieve maximum density, the sample production was carried out using the standard parameters provided by ARCAM. Subsequently, the built samples were cut from the 316L stainless steel base plate using a wire electric discharge machining machine and subjected to the thermal processes as outlined in the test plan presented in Table 1. For samples labeled as HT1, solely a solution treatment was conducted at 950 °C for 30 min, followed by furnace cooling after the treatment. For other heat-treated samples (from HT2 to HT6), the solution treatment was initially applied with the same parameters as those used for the HT1 sample, followed by subsequent aging processes. The aging parameters, including time, temperature, and cooling, were applied in accordance with the test plan. A group of samples underwent HIP processing with an AIP6-30H machine. In the HIP1 procedure, the lowest temperature (800 °C) and highest pressure (200 MPa) were utilized, while the HIP2 procedure employed a combination of a moderate temperature (920 °C) and low pressure (100 MPa). In the HIP3 procedure, the highest temperature (1050 °C) and a relatively lower pressure (100 MPa) were applied. All thermal processes, including solution treatment, aging, and HIP processes, were carried out under an argon atmosphere.

**Table 1:** HT and HIP Parameters Applied to the EBM Ti_6_Al_4_ V Sample^a^

samplecode	thermal process	time (min)	temperature (°C)	cooling	pressure (MPa)
EBM	—	—	—	—	—
HT1	ST	30	950	FC	—
HT2	(ST + AG)	240	500	AC	—
HT3	(ST + AG)	240	550	AC	—
HT4	(ST + AG)	600	500	AC	—
HT5	(ST + AG)	600	550	AC	—
HT6	(ST + AG)	600	600	AC	—
HIP1	HIP	120	800	FC (30 K/min)	200
HIP2	HIP	120	920	FC (30 K/min)	100
HIP3	HIP	120	1050	FC (30 K/min)	100

a ST = solution annealing treatment, AG = Aging, FC = furnace cooling, and AC = air cooling.

### 2.2 Microstructure Characterization

Microstructural investigation of samples was carried out using an optical microscope (Nikon Eclipse E200). Prior to metallographic examinations, the samples were initially ground using 1000 and 2500 grit SiC abrasive sandpapers, followed by polishing with 1 μm diamond paste. Finally, the samples were finished with a colloidal silica suspension. Following surface preparations, samples were chemically etched with a solution of 91 mL of water, 6 mL of HNO_3_, and 3 mL of HF (Kroll’s reagent). The quantitative analysis of the phases and density measurements was performed by using FIJI software package (free version). The α lath thickness was measured by the intersection method.^16^ The phase analysis was achieved qualitatively with a Rigaku Miniflex 600 X-ray diffractometer with CuKa radiation (λ = 1.540562 Å) operated at 40 kV and 15 mA, and a scanning rate of 2°/min. The corrosion area was imaged and semiquantitatively analyzed by field emission scanning electron microscopy (FESEM) model Hitachi Regulus 8230 FESEM and energy-dispersive spectroscopy (EDS) detector in the line mode, respectively.

### 2.3 Electrochemical Measurements

The Gamry-Interface model 1000 was employed for OCP, potentiodynamic polarization, and EIS tests. Prior to corrosion testing, the samples were cut in half using a Struers Secotom-15 metallographic cutting device and the tests were conducted on planes parallel to the build direction. Electrochemical measurements were performed in a 3.5 wt % NaCl solution (ASTM G44) using a three-electrode system consisting of a working electrode (sample), a reference electrode (saturated Ag/AgCl), and a counter electrode (carbon bare) at room temperature. To prepare the working electrode, a conductive electrical cable was attached to the Ti_6_Al_4_ V samples using adhesive copper tape. Subsequently, the Ti_6_Al_4_ V samples were cold-mounted with epoxy resin. The prepared sample was ground with 800 and 1200 rpm by using SiC adhesive papers in a water-cooled metallographic polisher/grinder. The finished surface was rinsed with distilled water and allowed open-air drying. Prior to all tests, the reference electrode was verified by measuring the impedance for proper functionality. The OCP test started after 15 min of immersion. The OCP (*E*_ocp_) was recorded for 2 h, and the results were presented as *E*_ocp_ (mV) versus time (*s*). The potentiodynamic polarization experiments were performed from −1000 to 7000 mV at a scan rate of 1 mV/s. The Tafel method was applied to polarization curves using the software Gamry Echem Analyst V7.05 to find the corrosion potential (*E*_cor_) and corrosion current density (*i*_corr_). The EIS technique was employed to assess the impedance resistance of the sample. The Nyquist curves were obtained at an amplitude of 10 mV in the frequency range of 10^5^ and 10^–2^ Hz. The spectra of EIS were evaluated by fitting a relevant electrical equivalent circuit (EEC) to Nyquist curves. All experiments were repeated at least three times at room temperature.

## 3 Results and Discussion

### 3.1 Microstructure Evaluation

**Figure 1:**
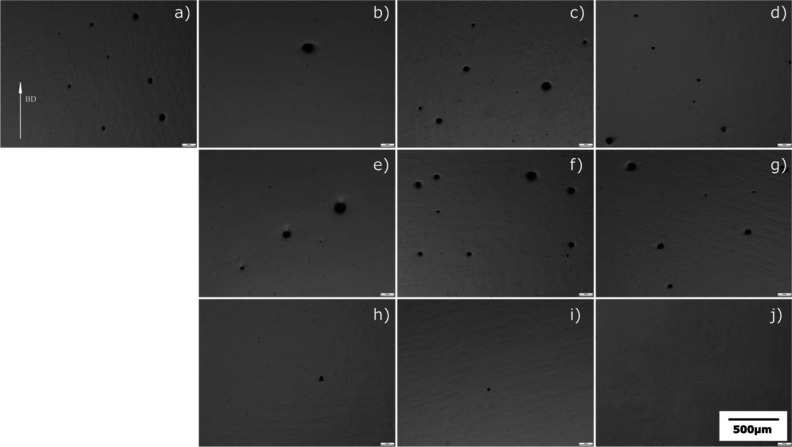
Optical images of samples showing the porosity on the surface; (a) as built (b) HT1, (c) HT2, (d) HT3, (e) HT4, (f) HT5, (g) HT6, (h) HIP1, (i) HIP2, and (j) HIP3 (BD: build direction).

**Figure 2:**
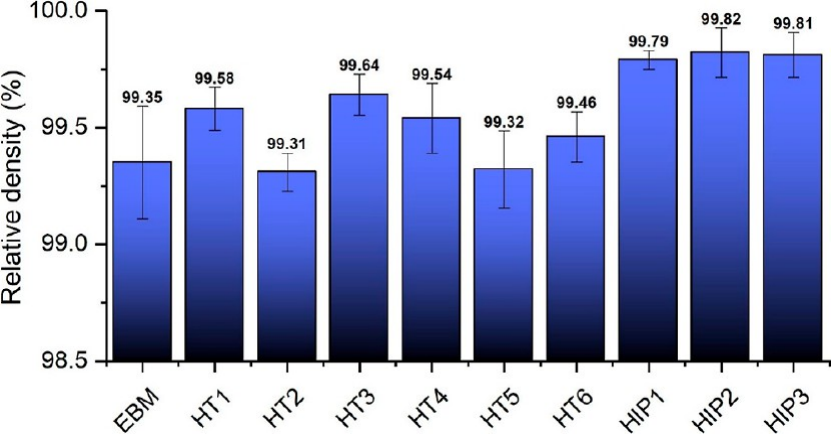
Relative density of EBM (as-built), HT, and HIP samples.

Figure 1 shows the OM images of as-built, heat treated, and hot isostatic pressed samples. It can be observed that both as-built and heat-treated samples exhibited a number of porosities. The morphology of the pores is generally circular, with diameters varying within a certain range. These porosities are probably associated with the stock material, which contains gas voids originating from the powder production process. During gas atomization, gas is trapped in the molten metal, resulting in the creation of spherical voids within the powder particles.^39^ The gas voids within the bulk material are distributed randomly, varying in size from 1 to 100 μm^3^. Even though HTs show minimal impact on porosity, the HIP processes are highly effective in nearly eradicating the pores entirely. The density measurement results of the samples, presented in Figure 2, confirm this trend. Analysis conducted using image processing techniques demonstrated a substantial increase in densities across all HIP methods.

**Figure 3:**
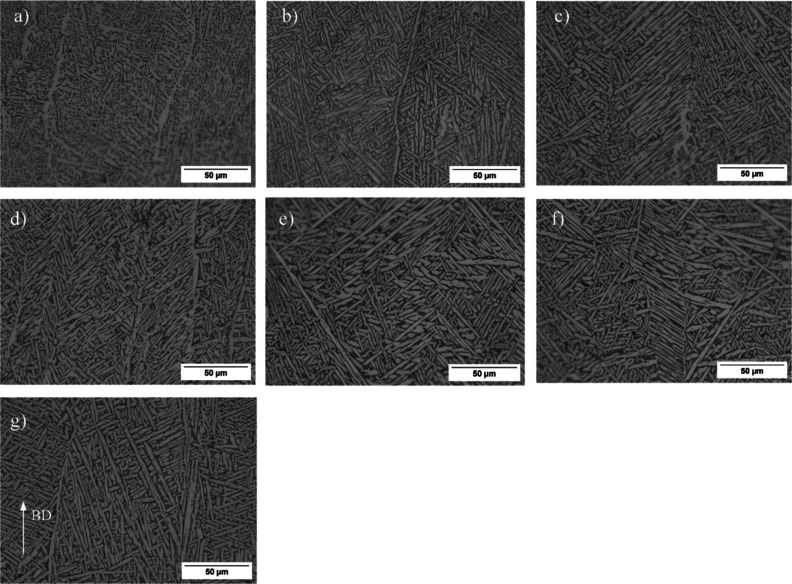
Optical images of the heat-treated samples; (a) as built, (b) HT1, (c) HT2, (d) HT3, (e) HT4, (f) HT5, and (g) HT6 (BD: build direction).

**Figure 4:**
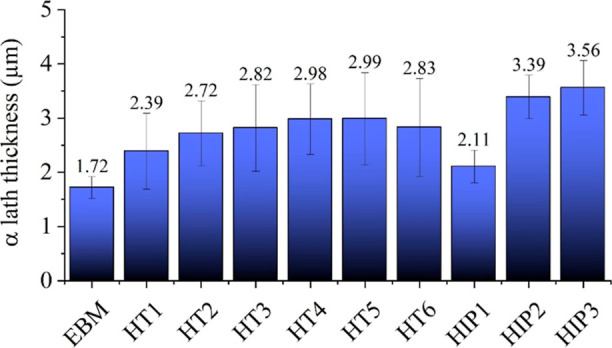
α-Lath thickness of EBM (as-built), HT, and HIP samples.

**Figure 5:**
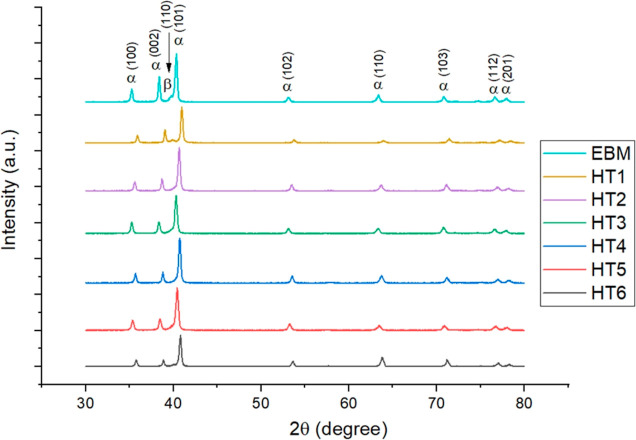
XRD patterns of EBM (as-built) sample vs HT samples.

**Figure 6:**
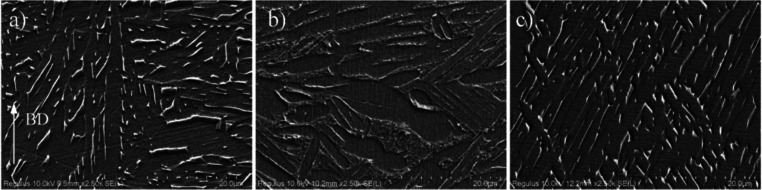
SEM images of (a) EBM (as-built) sample, (b) HT4 sample, and (c) HIP4 sample (BD: build direction).

It is well-known that high cooling rates and directional solidification in EBM parts lead to a columnar structure.^6^ The microstructures obtained from the as-built and thermally postprocessed samples are shown in Figure 3. The as-built sample exhibits columnar primary β structures, which are characteristic of AM processes, such as powder bed fusion and direct energy deposition techniques. The microstructure of the as-built sample consists of primary β columns and lamellar α + β structures within these primary β columns. Moreover, primary β grain boundaries are decorated with continuous grain boundary α_gb_ phases. The thickness of the α phase laths was measured using an image analysis method, and the results for various samples can be observed in Figure 4. The as-built sample had an average α-lath thickness of about 1.7 μm. The solution annealing HT has no remarkable effect on columnar structure as seen for the HT1 sample but increased the α-lath thickness to 2.3 μm due to the diffusion process. The aging treatment also led to a moderate increase in α-lath thickness for HT2 to HT6 samples (Figure 4). While the aging temperature had no significant effect on the resulting microstructure, which may be attributed to the small differences in aging temperatures, a slight increase in aging duration resulted in the coarsening of α-laths. All heat-treated and HIP-processed samples maintained the columnar microstructure, except for the samples treated with the HIP3 procedure at the highest temperature. Prior studies have demonstrated that implementing secondary thermal processes, such as HT or HIP, below the transus temperature maintains the columnar microstructure characteristic of the EBM process. Conversely, conducting HT or HIP processes above the transus temperature results in a transition from a columnar to an equiaxed morphology.^15,17,40,41^ X-ray diffraction (XRD) analysis revealed that the as-built samples exhibit α and β phase peaks simultaneously (Figure 5). In the solution annealed sample (HT1), the β phase peak is more pronounced, suggesting an increase in β phase content. However, the peak belonging to the β phase was not distinctive after aging according to Figure 5. This removal of the retained β phase and the increase in α phase during the aging process aligns with findings in other studies.^42−44^ The variation of the β-phase fraction is related to the diffusive transformation that occurs during aging. This process results in the decomposition of the retained β phase into a thermodynamically more stable microstructure, which contains a higher amount of α phase.^42^ To observe the effect of aging on the microstructure, high-resolution SEM images were obtained from both the as-built aged (HT4) and HIP (HIP4) samples. These images are displayed in Figure 6. In the as-built sample, typically rod- and dot-shaped bright β phases are visible in the microstructure. However, the HT4 sample displayed a lower amount of the brightly colored β phase. The β phase ratios measured from the SEM images showed that the as-built sample has a 7.5% β phase, whereas the HT4 sample has a 3.3% β phase. Moreover, in the as-built sample, the β phase rods are continuous, while in the HT4 sample, the rods are discontinuous, which can be interpreted as the nucleation of α phases within the β phases.

**Figure 7:**
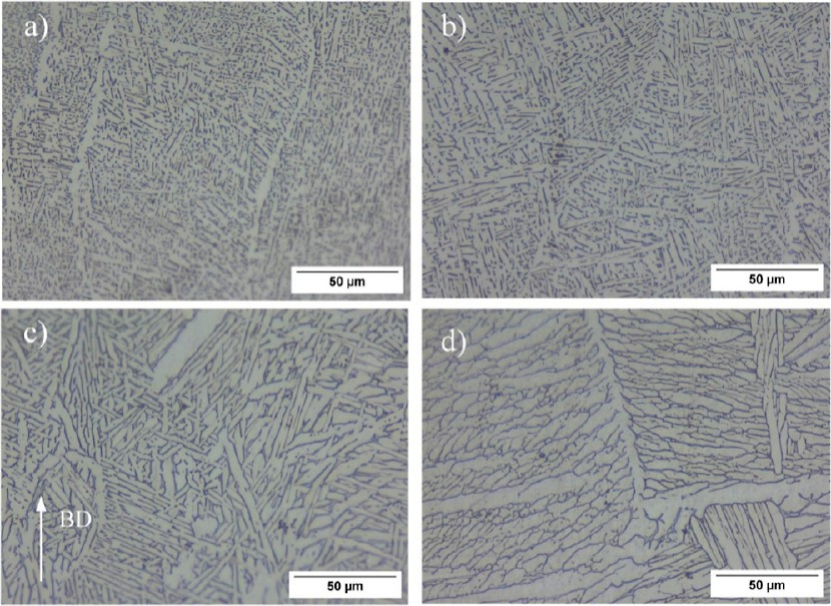
Optical images of the HIPed samples; (a) as built, (b) HIP1, (c) HIP2, and (d) HIP3 (BD: build direction).

**Figure 8:**
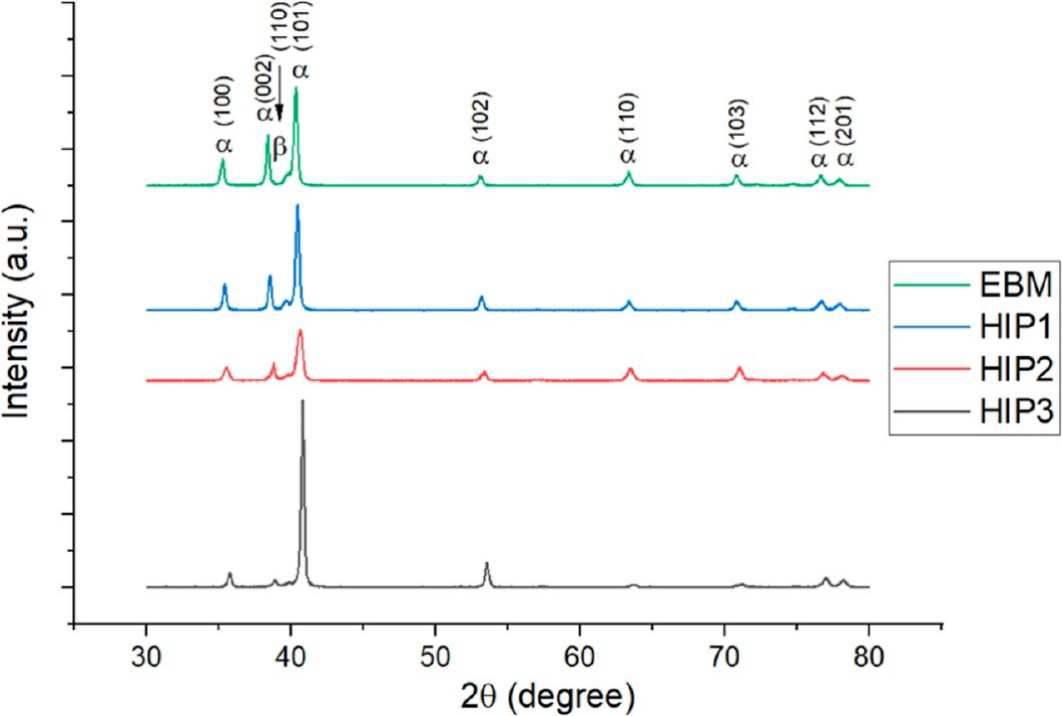
XRD patterns of the HIP process samples vs the EBM sample.

The HIP process also modified the microstructure of the Ti_6_Al_4_ V alloy as can be seen in Figure 7. Although the HIP1 process, characterized by low temperature and high pressure, resulted in an enlargement of α laths, this increase was relatively limited compared to other HIP cycles, primarily due to the lower temperature employed in the HIP1 cycle. It is clearly seen that the increasing HIP temperature to 920 °C caused a much more severe enlargement in α laths. Furthermore, formation of the α phase with a globular shape can be found locally in the microstructure. The HIP3 treatment (high temperature, low pressure) led to greater changes in the microstructure compared to the other HIP processes due to its supertransus temperature. The columnar primary β phases were recrystallized into equiaxed grains, and the α laths within the primary β grains became significantly coarser. In addition, it is seen that in the form of basket wave binary α + β structure (Widmanstätten morphology) has left its place to α colony dominated microstructure in the samples subjected to the HIP3 process. In samples subjected to HIP above the transus temperature, a noticeably thickened α_gb_ network on the primary β grain boundaries is clearly evident. The XRD results indicated that the HIP1 process resulted in a more pronounced β-phase peak, suggesting an increase in β-phase content (Figure 8). The SEM image of the HIP1 sample in Figure 6c showed that the typically rod- and dot-shaped bright-colored β phases inherited from EBM were preserved after the HIP1 procedure. Additionally, the β phase ratio measured from the image increased from 7.5 to 9.5% after HIP1 treatment, consistent with the XRD observations. Conversely, the XRD results for the HIP2 and HIP3 processes did not show a significant β phase peak. This may imply that HIP2 and HIP3 processes possibly reduced the β phase ratio.

### 3.2 Corrosion Behavior

**Figure 9:**
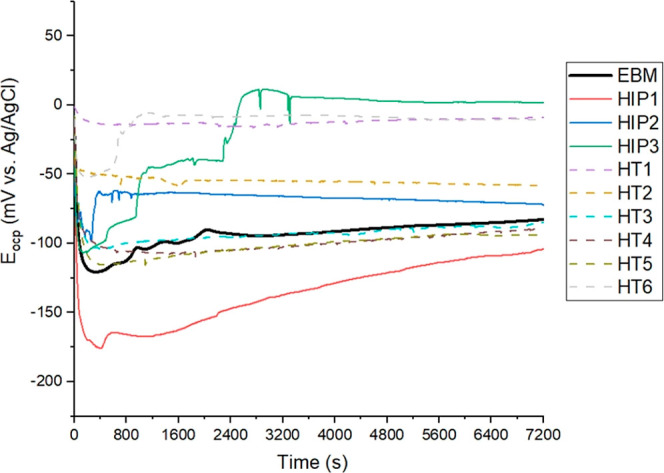
OCP behavior of samples after immersion in 3.5 wt % NaCl solution.

The OCP test provides a survey of the OCP (*E*_ocp_) behavior of the sample in the immersed solution. According to the literature, Ti_6_Al_4_ V forms a TiO_2_ or TiO_2_/Al2O_3_ protective oxide layer after immersion in a chloride environment.^22,45^ After 15 min of immersion in a 3.5 wt % NaCl solution, all samples exhibited a downward *E*_ocp_ trend for up to 1000 s, indicative of corrosion activity at the interface, as depicted in Figure 9. The *E*_ocp_ shifted positively after this period for both as-built and thermally treated samples, indicating the slow formation of the protective passive film, as observed in various studies.^22,26,46^ The *E*_ocp_ of the as-built EBM sample reached −82 mV after 2 h of immersion, which is approximately 312 and 368 mV more noble than the EBM-XY and EBM-YZ samples tested in a similar solution for 12 h, respectively.^28^ When comparing our results to the study conducted by Metalnikov et al.,^22^ our as-built EBM sample exhibited a more noble *E*_ocp_ compared to the EBM-XY (−270 mV) and EBM-XZ (−120 mV) samples, which were immersed for 50 h in a 0.9 M NaCl solution. The worst corrosion potential behavior of the EBM sample can be attributed to the heterogeneous distribution of iron elements in the β phase, which causes a galvanic effect with the α phase.^47^

When considering the corrosion potentials of thermally treated samples, it is evident that the HIP3 sample becomes more anodic after 2400 s compared to its counterparts after 2 h of immersion. Among the HIP-treated sample group, HIP3 exhibited a more noble corrosion potential compared with HIP2- and HIP1-treated samples. It reached approximately 0 mV with an *E*_ocp_ of 1.2 mV, which was about 74 and 104 mV more anodic compared to HIP2 and HIP1, respectively. Interestingly, the *E*_corr_ values of HIP2 and HIP3 samples exhibited spikes at certain periods, and the reasons for these phenomena remain unclear. This fluctuation has also been reported by various authors.^28,48^ On the other hand, the *E*_ocp_ of the HT1-treated sample displayed a superior corrosion potential when compared to the other HT samples, starting at approximately −0.2 mV and ending at −8.6 mV. The HT2, HT3, HT4, HT5, and HT6 samples were 50, 76, 80, 85, and 3 mV more cathodic compared to the HT1 sample, respectively. The initial findings suggest that certain HTs, such as HIP2, HIP3, HT1, HT2, and HT6, appear to improve the *E*_ocp_ behavior in a 3.5 wt % NaCl solution.

**Figure 10:**
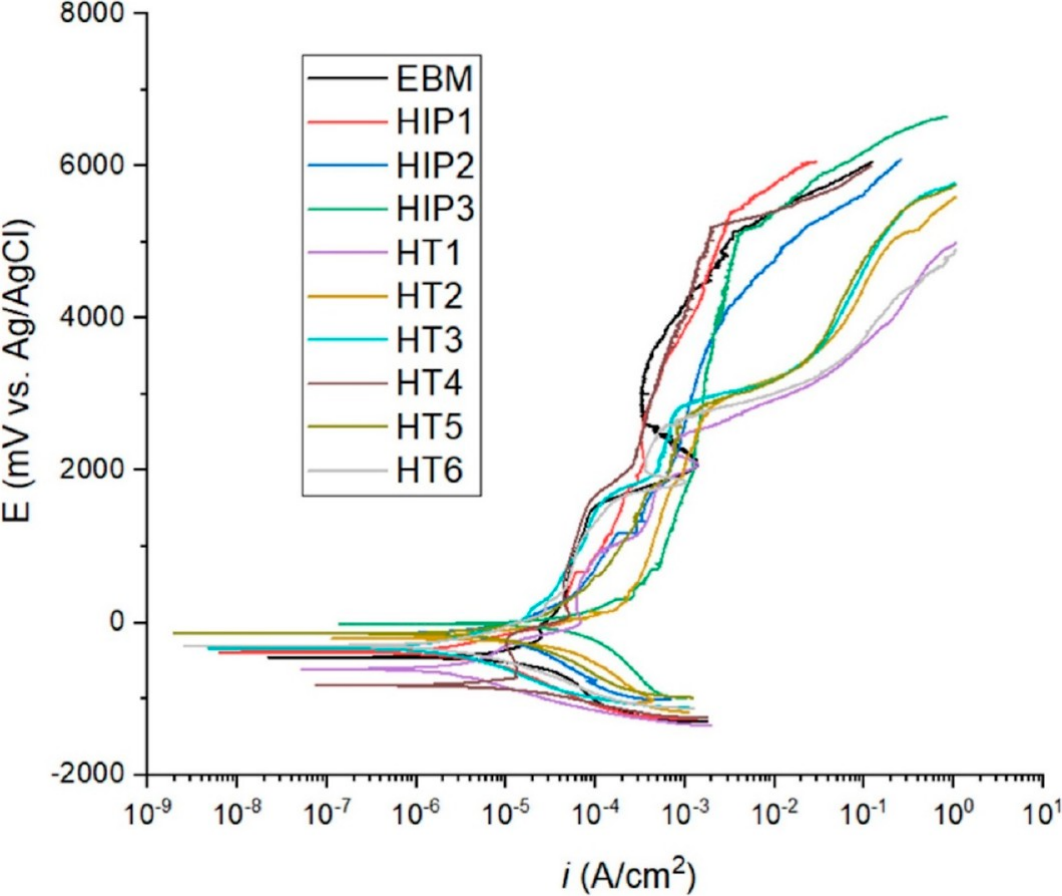
Potentiodynamic curves of samples obtained in 3.5 wt % NaCl solution.

The potendiodynamic polarization curves of EBM and heat-treated samples are illustrated in Figure 10 and the data obtained are presented in Table 2. Here, the corrosion potential (*E*_corr_) of the as-built sample measured was −454 mV with a current density of 13.2 μA. Among the HIP-treated sample, the HIP2 had a lower corrosion tendency with −136 mV compared to the HIP1 and HIP3 samples. It should be noted that the grain size as well as the phase distribution affect the corrosion resistance of metals. Although the HIPed samples had a slightly larger grain size distribution than the EBM sample, it appears that the equiaxed grain structure improved the corrosion potential. Regarding HT, the HT5 sample exhibited higher corrosion resistance at −141 mV. In contrast, the corrosion potential of HT4 was more cathodic at −818 mV compared to the as-built EBM sample. All the tested samples displayed an evident passivation zone in the 3.5 wt % NaCl solution. The EBM as-built sample displayed two passivation zones: the first one ranged from −142 to −24 mV, and the second one extended from 2122 to 3393 mV. After HIPing, the first passivation region was larger for HIP2 specimens. However, the second passivation was not observed for the same sample. Analysis of the second passivation region confirmed that HIP3 had a broader passivation zone, which was 954 mV greater than the as-built EBM sample. On the other hand, all the heat-treated samples exhibited similar behavior, except for HT1 and HT4, which displayed three passivation regions instead of two. This suggests the possibility of forming a passive layer in these regions.

**Table 2:** Tafel Data Are Obtained from Figure 10

sample	beta A (V/decade)	beta C (V/decade)	*i*_corr_ (μA)	E_corr_ (mV)	passivation #1 (mV)	passivation #2 (mV)	passivation #3 (mV)
EBM	7.98 × 10^–1^	3.33 × 10^–1^	13.2	–454	–142/–24	2122/3393	
HIP1	3.39 × 10^–1^	4.26 × 10^–1^	2.86	–380	92/362	1860/2350	
HIP2	9.53 × 10^–1^	8.60 × 10^–1^	17.2	–136	1183/1548		
HIP3	7.48 × 10^–1^	7.51 × 10^–1^	15.5	–237	860/1100	1129/3954	
HT1	4.67 × 10^–1^	3.73 × 10^–1^	2.60	–613	–27/250	1165/1739	2065/2425
HT2	4.35 × 10^–1^	5.70 × 10^–1^	21.0	–190	335/391	1950/2058	
HT3	4.34 × 10^–1^	3.79 × 10^–1^	1.96	–331	148/174		
HT4	2.33 × 10^–1^	2.34 × 10^–1^	9.85	–818	–712/–683	–671/–229	1580/1605
HT5	9.66 × 10^–1^	7.19 × 10^–1^	19.1	–141	612/668	1850/2680	
HT6	4.43 × 10^–1^	3.55 × 10^–1^	2.84	–304	181/293	1872/2576	

When comparing the passivation regions, HT1 had a larger passive layer in the first and third regions, while HT5 had a larger passive layer in the second region. The current density value or corrosion rate was calculated by Tafel plots and is given in Table 2. At first glance, it appears that the current density of HT3 in the HT-treated group and HIP1 in the HIP-treated group was lower, suggesting reduced ion exchange. In contrast, the current passage was higher for HIP2 and HT2 compared to the EBM as-built specimen.

**Figure 11:**
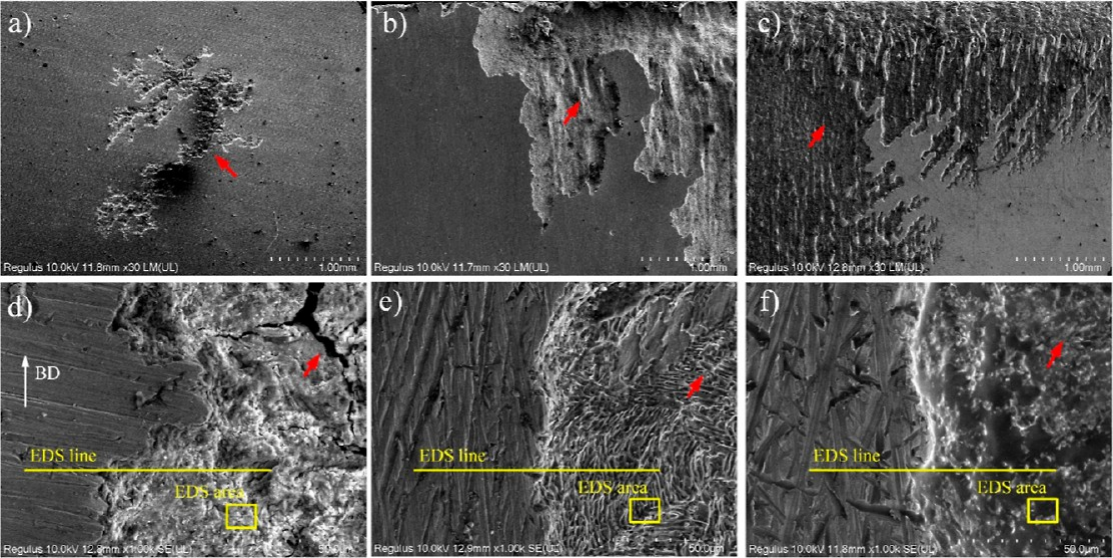
FESEM images showing the corroded surface after polarization test (red arrow showing the corroded area); (a) x30 EBM, (b) x30 HT1, (c) x30 HIP1, (d) x1000 EBM, (e) x1000 HT1, and (f) x1000 HIP1 (BD: build direction).

**Figure 12:**
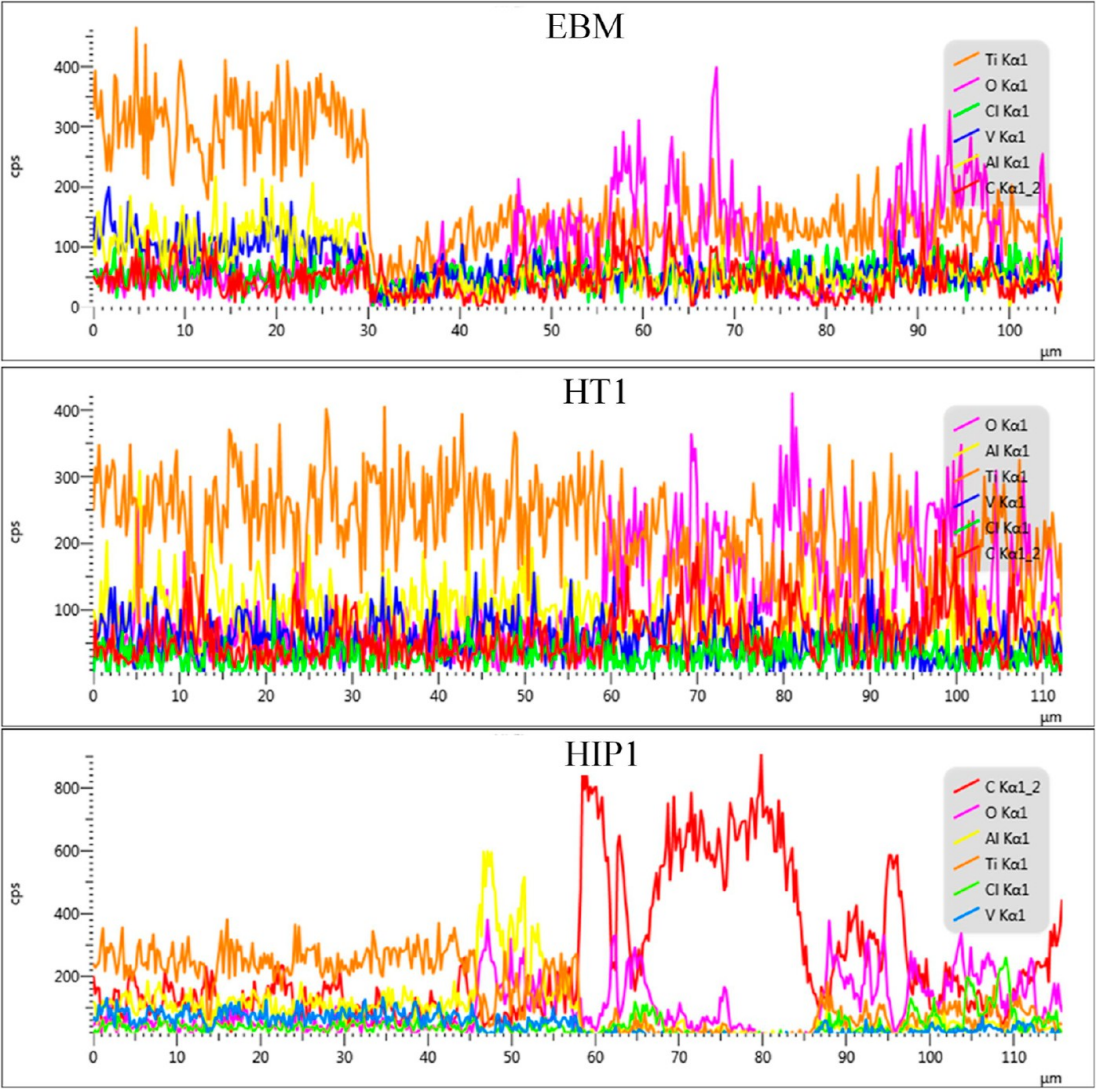
FESEM–EDS line results from Figure 11 showing the element variation.

The corroded surface of untreated and treated samples was examined after potentiodynamic tests, and the images are presented in Figure 11. One sample from each group was analyzed due to their similar corrosion behavior. Here, it can be stated that the corrosion was localized and that the pits progressed within the Widmanstätten microstructure for the EBM as-built sample (Figure 11a–d). As reported, the Widmanstätten microstructure for EMB-Ti_6_Al_4_ V is composed of two phases; α-needle like and β- compromised between α-needle like.^7,28^ This microstructure had better corrosion resistance compared to equiaxed microstructure for wrought Ti_6_Al_4_ V alloy.^49^ On the other hand, the heterogeneous distribution of Fe which is the alloying element could also affect the corrosion resistance of EBM Ti_6_Al_4_ V.^28^ Furthermore, the building direction affects the corrosion performance of AM Ti_6_Al_4_ V alloys. It was revealed that the building direction 0° had better corrosion resistance because of low β phase quantity.^27^ The EDS line analysis of the corroded surface confirms a sudden decrease in the quantity of Ti, Al, and V elements at the pitting region and the formation of oxide particles originating from corrosion products (Figure 12). The chemical elements found in the corrosion area are given in Table 3. It appears that the quantity of titanium oxide is higher compared to that of aluminum and vanadium oxides. Concerning the HT- and HIP-treated samples, the corrosion was more uniform instead of localized for HT1 (Figure 11b–e) and HIP1 (Figure 11c–f). Furthermore, the β-phase is clearly visible in the corroded region of the HT1 sample due to the HT process (Figure 11e). It should be noted that the presence of the β-phase was also found on XRD peaks for HT1 (Figure 5). However, the analysis of the uncorroded and corroded region with EDS line showed increase of oxygen suggesting formation of oxide corrosion product. In adverse, a slight decrease of the Ti, Al, and V element concentration was spotted except that C was at some region increased (Figure 12). This may be explained by the presence of the β-phase and continuous grain boundary α_gb_ phases, which act as protective barriers against corrosion degradation. According to Xiu et al., both α and β phases had almost similar composition, and β-phase had slightly higher V and Fe, while the α-phase had higher Al element.^34^ Regrading to the EDS area of HT1, the V and Ti were higher compared to corroded area of EBM (Table 3), which may explain the better corrosion resistance of HT1 versus EBM.

**Table 3:** FESEM–EDS Area Analysis Performed on the Corrosion Area at Figure 11

element	EBM (wt %)	HT1 (wt %)	HIP1 (wt %)
C	6.26	9.15	31.15
O	26.57	18.65	13.36
Al	3.85	3.64	1.76
Cl	3.71	1.44	11.35
Ti	58.37	64.25	39.05
V	1.24	2.88	3.33
total	100	100	100

On the other hand, it was noticed that the corrosion continued in a columnar form for HIP1, similar to what was observed in the EBM sample (Figure 11c–f). Furthermore, the presence of the β-phase (white color) with the α-phase was observed in the corroded area. The semiquantitative analysis with EDS line elucidated a sudden increase of aluminum and oxygen at the intersection of uncorroded and corroded region. Nevertheless, Ti and V element concentration decreased in the corroded area (Figure 12). The increase in the Al element can indicate the high presence of the α-lath phase as reported in the literature.^34^ Meanwhile, it was noted that the pitting corrosion occurs predominantly at the α-phase, while the β-phase seems more resistant against corrosion for EBM Ti_6_Al_4_ V. On the other hand, carbon quantity was found higher in the corroded area for HIP1 (Figure 12 and Table 3). The carbon-alloying element is used as α-stabilizing element such as Al, O, and N. It can be deduced from this finding that the α-phase was more pronounced in the corroded area than β-phase.

**Figure 13:**
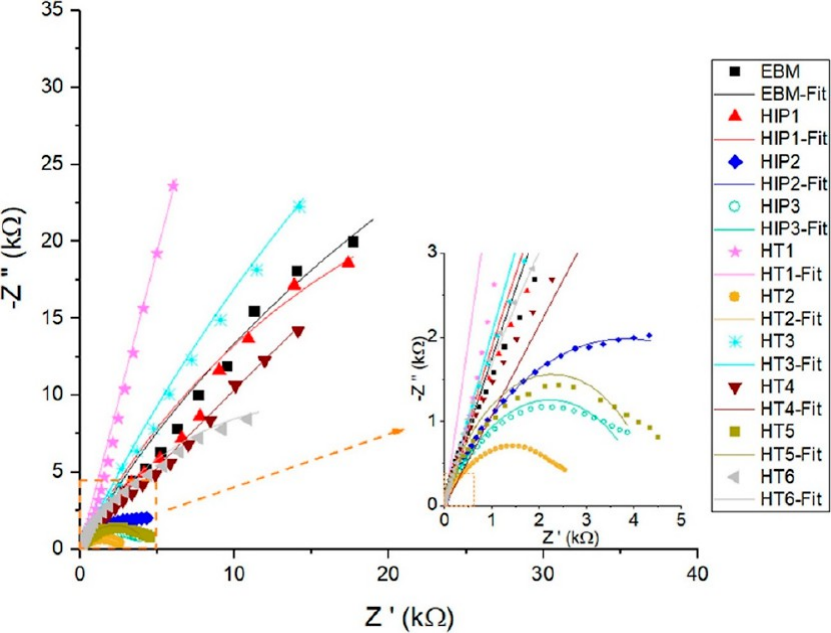
Nyquist curve of samples collected in 3.5 wt % NaCl solution.

The properties of the protective oxide layer of heat-treated samples were investigated by EIS measurement. The Nyquist plots are given in Figure 13 with the EEC fitting plots. At first glance, all samples exhibited a capacitive loop at high frequency, indicating a resistance of the surface against corrosion. Additionally, HIP2 exhibited a medium capacitive loop, while HT2, HT5, and HIP3 showed an incomplete capacitive loop at low frequency.

**Figure 14:**
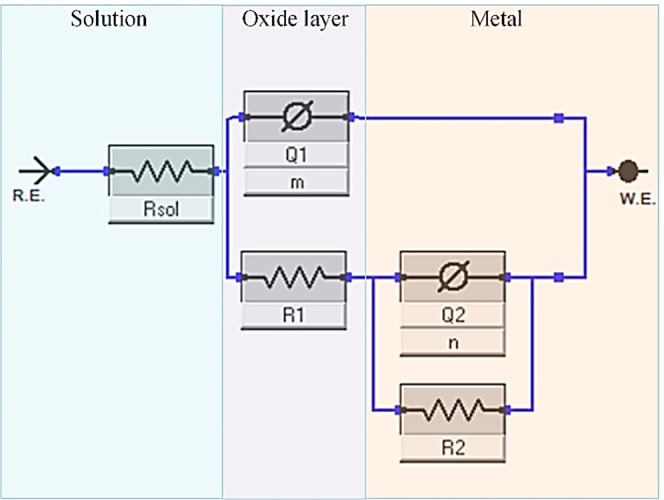
Equivalent circuit model of the samples.

The plots were fitted to the one-time constant circuit, as presented in Figure 14. This circuit was chosen because the 3.5 wt % solution was considered aggressive. In this circuit, the elements *R*_sol_, *R*_1_, and *R*_2_ of the EEC correspond to the solution resistance, oxide layer, and charge transfer resistance, respectively. The fitted results of R1 revealed that the HT HT1 sample had an excellent passivity in the 3.5 wt % NaCl compared to the other samples, which is in good agreement with the findings of Tafel study (see Table 2). According to the literature,^45^ a larger radius loops represent better resistance of the protective oxide layer against corrosion. Based on this consideration, the spectra of HT1 had larger radii when compared to those of its counterparts. This improvement can be attributed to (i) the presence of a coarser α-lath phase and (ii) a well-defined β phase, which likely acted as barriers during corrosion. Conversely, the lowest *R*_1_ value was observed for the HIP1 sample, and this can be attributed to the larger grain size distribution and the presence of the β phase. This worse corrosion behavior of HIP1 was also observed during *E*_ocp_ measurement, where the measured *E*_ocp_ after 2 h was more cathodic than the as-built EBM, HT, and HIP samples (see Figure 9). Regarding the charge transfer (*R*_2_) resistance at the interface between the oxide layer and the metal, it was higher for HT4 and lower for HIP2. These results are consistent with the *E*_corr_ behavior of HT4 and HIP2 in Figure 10; HT4 had more cathodic, while HIP2 had more anodic corrosion potential (see Table 2). Furthermore, it was reported that conventional Ti_6_Al_4_ V exhibited better charge transfer resistance than AM-manufactured Ti_6_Al_4_ V, regardless of the sample plane, in a 3.5 wt % NaCl solution.^50^

**Table 4:** Data Were Extracted after Fitting the Nyquist Curves in Figure 13 with the ECC Model Presented at Figure 14

sample	*R*_sol_ (ohms)	*R*_1_ (ohms)	*R*_2_ (ohms)	*Q*_1_ (S ×s^a^)	m	*Q*_2_ (S ×s^a^)	N	goodness of fit
EBM	1.212	1.75 × 10^4^	1.00 × 10^5^	1.90 × 10^–4^	6.80 × 10^–1^	9.63 × 10^–4^	9.96 × 10^–1^	1.68 × 10^–2^
HIP1	1.368	1.01 × 10^2^	8.27 × 10^4^	2.20 × 10^–4^	7.04 × 10^–1^	1.10 × 10^–5^	1	1.40 × 10^–2^
HIP2	1.792	7.74 × 10^3^	1.45 × 10^2^	1.24 × 10^–4^	8.78 × 10^–1^	4.11 × 10^–4^	5.59 × 10^–1^	2.59 × 10^–4^
HIP3	1.030	3.43 × 10^3^	8.81 × 10^2^	2.06 × 10^–4^	8.30 × 10^–1^	4.17 × 10^–4^	6.40 × 10^–1^	2.03 × 10^–3^
HT1	1.657	4.10 × 10^8^	7.73 × 10^3^	4.10 × 10^–4^	8.39 × 10^–1^	1.31 × 10^–11^	7.65 × 10^–1^	8.86 × 10^–3^
HT2	1.406	2.80 × 10^3^	2.42 × 10^2^	1.05 × 10^–4^	8.10 × 10^–1^	3.34 × 10^–4^	4.95 × 10^–1^	3.14 × 10^–4^
HT3	1.671	3.30 × 10^2^	2.02 × 10^5^	2.51 × 10^–4^	7.17 × 10^–1^	9.25 × 10^–5^	1.62 × 10^–2^	1.07 × 10^–2^
HT4	1.683	1.27 × 10^2^	5.02 × 10^5^	2.10 × 10^–4^	5.25 × 10^–1^	1.80 × 10^–8^	2.50 × 10^–1^	1.34 × 10^–2^
HT5	1.611	3.34 × 10^3^	1.17 × 10^3^	3.09 × 10^–4^	8.79 × 10^–1^	3.49 × 10^–4^	6.98 × 10^–1^	2.75 × 10^–3^
HT6	1.888	5.31 × 10^3^	3.07 × 10^4^	3.21 × 10^–4^	6.95 × 10^–1^	1.08	5.00 × 10^–1^	3.53 × 10^–3^

In addition, constant phase elements *Q*_1_ and *Q*_2_ were used to fit the plot due to the inhomogeneity of the surface, such as roughness and porosity. When the values in Table 4, it becomes evident that the HT1 sample had a superior protective layer with low porosity compared to all the other samples. Meanwhile, HIP1 also exhibited improved corrosion resistance within the group of HIP samples. Additionally, both HT1 and HIP1 treatments resulted in lower roughness when comparing the *Q*_2_ values.

## 4 Conclusions

The Ti_6_Al_4_ V specimens produced via the EBM process underwent various HTs and HIP cycles to enhance corrosion resistance. Based on the findings, the thermal process parameters affect the microstructure and therefore the corrosion behavior of EBM parts. In summary, the following conclusions can be drawn from this study:

- The HT with aging process led to the disappearance of the β phase in the obtained microstructure. A similar observation was made when increasing the temperature and decreasing the pressure during HIPing. Additionally, both thermal processes resulted in an increase in the α-lath thickness.
- The *E*_ocp_ of HIP3 (supertransus temperature) was more anodic compared to other samples. In addition, wider passivation region formation was observed during anodic test. The impedance resistance test proved a larger capacitive loop implying a better oxide film resistance. This can be explained by the equiaxed grain structure with a coarser columnar structure/higher α-lath thickness and absence of the β phase.
- In the HT group, the *E*_ocp_ of HT1, which only underwent solution annealing, exhibited superior corrosion resistance compared to the other HT samples and the as-built one. Furthermore, anodic polarization revealed three passivation zones, with two of them being wider. This was also confirmed by the larger radius obtained with impedance test. This enhanced corrosion resistance can be attributed to the coarser α phase and well-defined β phase, which act as effective corrosion barriers. On the other hand, it was observed that the corrosion resistance of the solution-annealed and aged HT1 sample was superior to that of the as-built EBM sample.
- The imaging of the corroded area suggested pit formation for EBM, while corrosion was uniform for the HT and HIP samples. The corrosion progress was lamellar for HT and HIP. The presence of the β phase was observed in the corroded region for HT1 and HIP1 samples.

## References

[ref1] Andersson L.-E.; Larsson M. Device and Arrangement for Producing a Three-Dimensional Object. EP 2173538 B1, 2009.

[ref2] Milewski J. O. Additive Manufacturing of Metals From Fundamental Technology to Rocket Nozzles, Medical Implants, and Custom Jewelry; Springer Series in Materials Science; Springer, 2017; Vol. 258. http://www.springer.com/series/856.

[ref3] Liu S.; Shin Y. C. Additive Manufacturing of Ti_6_Al_4_V Alloy: A Review. Mater. Des. 2019, 164, 107552 10.1016/j.matdes.2018.107552.

[ref4] Suntharavel Muthaiah V. M.; Rajput M.; Tripathi A.; Suwas S.; Chatterjee K. Electrophoretic Deposition of Nanocrystalline Calcium Phosphate Coating for Augmenting Bioactivity of Additively Manufactured Ti-6Al-4V. ACS Mater. Au 2022, 2 (2), 132–142. 10.1021/acsmaterialsau.1c00043. 36855763 PMC9888615

[ref5] Lütjering G.; Williams J. C. Titanium; Springer: Berlin Heidelberg, New York, 2007.

[ref6] Al-Bermani S. S.; Blackmore M. L.; Zhang W.; Todd I. The Origin of Microstructural Diversity, Texture, and Mechanical Properties in Electron Beam Melted Ti-6Al-4V. Metall. Mater. Trans. A 2010, 41, 3422–3434. 10.1007/s11661-010-0397-x.

[ref7] Tan X.; Kok Y.; Tan Y. J.; Descoins M.; Mangelinck D.; Tor S. B.; Leong K. F.; Chua C. K. Graded Microstructure and Mechanical Properties of Additive Manufactured Ti-6Al-4V via Electron Beam Melting. Acta Mater. 2015, 97, 1–16. 10.1016/j.actamat.2015.06.036.

[ref8] Donachie M. J. Titanium: A Technical Guide; ASM International, 2000.

[ref9] DebRoy T.; Wei H. L.; Zuback J. S.; Mukherjee T.; Elmer J. W.; Milewski J. O.; Beese A. M.; Wilson-Heid A. d.; De A.; Zhang W. Additive Manufacturing of Metallic Components-Process, Structure and Properties. Prog. Mater. Sci. 2018, 92, 112–224. 10.1016/j.pmatsci.2017.10.001.

[ref10] Kok Y.; Tan X. P.; Wang P.; Nai M. L. S.; Loh N. H.; Liu E.; Tor S. B. Anisotropy and Heterogeneity of Microstructure and Mechanical Properties in Metal Additive Manufacturing: A Critical Review. Mater. Des. 2018, 139, 565–586. 10.1016/j.matdes.2017.11.021.

[ref11] Yan M.; Yu P. An Overview of Densification, Microstructure and Mechanical Property of Additively Manufactured Ti-6Al-4V—Comparison among Selective Laser Melting, Electron Beam Melting, Laser Metal Deposition and Selective Laser Sintering, and with Conventional Powder; IntechOpen, 2015.

[ref12] Zhang L.; Liu Y.; Li S.; Hao Y. Additive Manufacturing of Titanium Alloys by Electron Beam Melting: A Review. Adv. Eng. Mater. 2018, 20 (5), 1700842 10.1002/adem.201700842.

[ref13] Wang M.; Li H. Q.; Lou D. J.; Qin C. X.; Jiang J.; Fang X. Y.; Guo Y. B. Microstructure Anisotropy and Its Implication in Mechanical Properties of Biomedical Titanium Alloy Processed by Electron Beam Melting. Mater. Sci. Eng. A 2019, 743, 123–137. 10.1016/j.msea.2018.11.038.

[ref14] Raghavan S.; Nai M. L. S.; Wang P.; Sin W. J.; Li T.; Wei J. Heat Treatment of Electron Beam Melted (EBM) Ti-6Al-4V: Microstructure to Mechanical Property Correlations. Rapid Prototyp. J. 2018, 24 (4), 774–783. 10.1108/RPJ-05-2016-0070.

[ref15] Draelos L.; Nandwana P.; Srivastava A. Implications of Post-Processing Induced Microstructural Changes on the Deformation and Fracture Response of Additively Manufactured Ti-6Al-4V. Mater. Sci. Eng. A 2020, 795, 139986 10.1016/j.msea.2020.139986.

[ref16] Galarraga H.; Warren R. J.; Lados D. A.; Dehoff R. R.; Kirka M. M.; Nandwana P. Effects of Heat Treatments on Microstructure and Properties of Ti-6Al-4V ELI Alloy Fabricated by Electron Beam Melting (EBM). Mater. Sci. Eng. A 2017, 685, 417–428. 10.1016/j.msea.2017.01.019.

[ref17] Syed A. K.; Awd M.; Walther F.; Zhang X. Microstructure and Mechanical Properties of As-Built and Heat-Treated Electron Beam Melted Ti-6Al-4V. Mater. Sci. Technol. 2019, 35 (6), 653–660. 10.1080/02670836.2019.1580434.

[ref18] Abdeen D. H.; Palmer B. R. Corrosion Evaluation of Ti-6Al-4V Parts Produced with Electron Beam Melting Machine. Rapid Prototyp. J. 2016, 22 (2), 322–329. 10.1108/RPJ-09-2014-0104.

[ref19] Yang J.; Yang H.; Yu H.; Wang Z.; Zeng X. Corrosion Behavior of Additive Manufactured Ti-6Al-4V Alloy in NaCl Solution. Metall. Mater. Trans. A 2017, 48, 3583–3593. 10.1007/s11661-017-4087-9.

[ref20] Shahsavari M.; Imani A.; Setavoraphan A.; Schaller R. F.; Asselin E. Electron Beam Surface Remelting Enhanced Corrosion Resistance of Additively Manufactured Ti-6Al-4V as a Potential in-Situ Re-Finishing Technique. Sci. Rep. 2022, 12 (1), 11589 10.1038/s41598-022-14907-2. 35804164 PMC9270471

[ref21] Kolamroudi M. K.; Asmael M.; Ilkan M.; Kordani N. Developments on Electron Beam Melting (EBM) of Ti-6Al-4V: A Review. Trans. Indian Inst. Met. 2021, 74, 783–790. 10.1007/s12666-021-02230-9.

[ref22] Metalnikov P.; Ben-Hamu G.; Eliezer D. Corrosion Behavior of AM-Ti-6Al-4V: A Comparison between EBM and SLM. Prog. Addit. Manuf. 2022, 7 (3), 509–520. 10.1007/s40964-022-00293-8.

[ref23] Szymczyk-Ziółkowska P.; Hoppe V.; Gąsiorek J.; Rusińska M.; Kęszycki D.; Szczepański Ł.; Dudek-Wicher R.; Detyna J. Corrosion Resistance Characteristics of a Ti-6Al-4V ELI Alloy Fabricated by Electron Beam Melting after the Applied Post-Process Treatment Methods. Biocybern. Biomed. Eng. 2021, 41 (4), 1575–1588. 10.1016/j.bbe.2021.10.002.

[ref24] Costa I.; Franco C. V.; Kunioshi C. T.; Rossi J. L. Corrosion Resistance of Injection-Molded 17–4PH Steel in Sodium Chloride Solution. Corrosion 2006, 62 (4), 357–365. 10.5006/1.3280667.

[ref25] Yeganeh M.; Shoushtari M. T.; Khanjar A. T.; Al Hasan N. H. J. Microstructure Evolution, Corrosion Behavior, and Biocompatibility of Ti-6Al-4V Alloy Manufactured by Electron Beam Melting (EBM) Technique. Colloids Surf., A 2023, 679, 132519 10.1016/j.colsurfa.2023.132519.

[ref26] Chen L. Y.; Huang J. C.; Lin C. H.; Pan C. T.; Chen S. Y.; Yang T. L.; Lin D. Y.; Lin H. K.; Jang J. S. C. Anisotropic Response of Ti-6Al-4V Alloy Fabricated by 3D Printing Selective Laser Melting. Mater. Sci. Eng. A 2017, 682, 389–395. 10.1016/j.msea.2016.11.061.

[ref27] Gong X.; Cui Y.; Wei D.; Liu B.; Liu R.; Nie Y.; Li Y. Building Direction Dependence of Corrosion Resistance Property of Ti-6Al-4V Alloy Fabricated by Electron Beam Melting. Corros. Sci. 2017, 127, 101–109. 10.1016/j.corsci.2017.08.008.

[ref28] Dehnavi V.; Henderson J. D.; Dharmendra C.; Amirkhiz B. S.; Shoesmith D. W.; Noël J. J.; Mohammadi M. Corrosion Behaviour of Electron Beam Melted Ti6Al4V: Effects of Microstructural Variation. J. Electrochem. Soc. 2020, 167 (13), 131505 10.1149/1945-7111/abb9d1.

[ref29] Gai X.; Bai Y.; Li J.; Li S.; Hou W.; Hao Y.; Zhang X.; Yang R.; Misra R. D. K. Electrochemical Behaviour of Passive Film Formed on the Surface of Ti-6Al-4V Alloys Fabricated by Electron Beam Melting. Corros. Sci. 2018, 145, 80–89. 10.1016/j.corsci.2018.09.010.

[ref30] Bai Y.; Gai X.; Li S.; Zhang L.-C.; Liu Y.; Hao Y.; Zhang X.; Yang R.; Gao Y. Improved Corrosion Behaviour of Electron Beam Melted Ti-6Al-4V Alloy in Phosphate Buffered Saline. Corros. Sci. 2017, 123, 289–296. 10.1016/j.corsci.2017.05.003.

[ref31] Nguyen H. D.; Pramanik A.; Basak A. K.; Dong Y.; Prakash C.; Debnath S.; Shankar S.; Jawahir I. S.; Dixit S.; Buddhi D. A Critical Review on Additive Manufacturing of Ti-6Al-4V Alloy: Microstructure and Mechanical Properties. J. Mater. Res. Technol. 2022, 18 (4641), 4641–4661. 10.1016/j.jmrt.2022.04.055.

[ref32] Zhao B.; Wang H.; Qiao N.; Wang C.; Hu M. Corrosion Resistance Characteristics of a Ti-6Al-4V Alloy Scaffold That Is Fabricated by Electron Beam Melting and Selective Laser Melting for Implantation in Vivo. Mater. Sci. Eng. C 2017, 70, 832–841. 10.1016/j.msec.2016.07.045. 27770961

[ref33] Carrozza A.; Cabrini M.; Lorenzi S.; Lombardi M.; Pastore T. Improving the Corrosion Performance of LPBF-and EBM-Processed Ti-6Al-4V by Chemical Pickling. Eng. Sci. 2023, 26, 985 10.30919/es985.

[ref34] Xiu M.; Tan Y. T.; Raghavan S.; Goh M. H.; Nai M. L. S. The Effect of Heat Treatment on Microstructure, Microhardness, and Pitting Corrosion of Ti6Al4V Produced by Electron Beam Melting Additive Manufacturing Process. Int. J. Adv. Manuf. Technol. 2022, 120 (1–2), 1281–1293. 10.1007/s00170-022-08839-4.

[ref35] Szymczyk-Ziółkowska P.; Ziółkowski G.; Hoppe V.; Rusińska M.; Kobiela K.; Madeja M.; Dziedzic R.; Junka A.; Detyna J. Improved Quality and Functional Properties of Ti-6Al-4V ELI Alloy for Personalized Orthopedic Implants Fabrication with EBM Process. J. Manuf. Process. 2022, 76, 175–194. 10.1016/j.jmapro.2022.02.011.

[ref36] Hosseini A. M.; Masood S. H.; Fraser D.; Jahedi M. Mechanical Properties Investigation of HIP and As-Built EBM Parts. Adv. Mater. Res. 2012, 576, 216–219. 10.4028/www.scientific.net/AMR.576.216.

[ref37] Tosi R.; Leung C. L. A.; Tan X.; Muzangaza E.; Attallah M. M. Revealing the Microstructural Evolution of Electron Beam Powder Bed Fusion and Hot Isostatic Pressing Ti-6Al-4V in-Situ Shelling Samples Using X-Ray Computed Tomography. Addit. Manuf. 2022, 57, 102962 10.1016/j.addma.2022.102962.

[ref38] Leon A.; Levy G. K.; Ron T.; Shirizly A.; Aghion E. The Effect of Hot Isostatic Pressure on the Corrosion Performance of Ti-6Al-4 V Produced by an Electron-Beam Melting Additive Manufacturing Process. Addit. Manuf. 2020, 33, 101039 10.1016/j.addma.2020.101039.

[ref39] Bauereiß A.; Scharowsky T.; Körner C. Defect Generation and Propagation Mechanism during Additive Manufacturing by Selective Beam Melting. J. Mater. Process. Technol. 2014, 214 (11), 2522–2528. 10.1016/j.jmatprotec.2014.05.002.

[ref40] Shui X.; Yamanaka K.; Mori M.; Nagata Y.; Kurita K.; Chiba A. Effects of Post-Processing on Cyclic Fatigue Response of a Titanium Alloy Additively Manufactured by Electron Beam Melting. Mater. Sci. Eng. A 2017, 680, 239–248. 10.1016/j.msea.2016.10.059.

[ref41] Galarraga H.; Lados D. A.; Dehoff R. R.; Kirka M. M.; Nandwana P. Effects of the Microstructure and Porosity on Properties of Ti-6Al-4V ELI Alloy Fabricated by Electron Beam Melting (EBM). Addit. Manuf. 2016, 10, 47–57. 10.1016/j.addma.2016.02.003.

[ref42] Carrozza A.; Marchese G.; Saboori A.; Bassini E.; Aversa A.; Bondioli F.; Ugues D.; Biamino S.; Fino P. Effect of Aging and Cooling Path on the Super β-Transus Heat-Treated Ti-6Al-4V Alloy Produced via Electron Beam Melting (EBM). Materials 2022, 15 (12), 4067 10.3390/ma15124067. 35744126 PMC9229345

[ref43] Huang J.; Yang Y.; Wang X.; Liang X.; Fu Y. Multi-Dimensional Effect of Heat Treatment on Microstructure and Property of Ti6Al4V Alloy Fabricated by Selective Electron Beam Melting. Metall. Mater. Trans. A 2022, 53 (9), 3357–3368. 10.1007/s11661-022-06750-x.

[ref44] Yang Y.; He J.; Huang J. Effect of Heat Treatment on Adiabatic Shear Susceptibility of Ti-6Al-4V Titanium Alloy Manufactured by Selective Electron Beam Melting. Mater. Sci. Eng. A 2022, 851, 143647 10.1016/j.msea.2022.143647.

[ref45] Pat S.; Hayati Çakir F.; Özgür Öteyaka M. Corrosion Behavior of Graphene Coated Ti-6Al-4 V Alloy by Anodic Plasma Coating Method. Inorg. Chem. Commun. 2023, 147, 110268 10.1016/j.inoche.2022.110268.

[ref46] Dai N.; Zhang L.-C.; Zhang J.; Zhang X.; Ni Q.; Chen Y.; Wu M.; Yang C. Distinction in Corrosion Resistance of Selective Laser Melted Ti-6Al-4V Alloy on Different Planes. Corros. Sci. 2016, 111, 703–710. 10.1016/j.corsci.2016.06.009.

[ref47] He X.; Noël J. J.; Shoesmith D. W. Effects of Iron Content on Microstructure and Crevice Corrosion of Grade-2 Titanium. Corrosion 2004, 60 (4), 378–386. 10.5006/1.3287747.

[ref48] Dai N.; Zhang L.-C.; Zhang J.; Chen Q.; Wu M. Corrosion Behavior of Selective Laser Melted Ti-6Al-4 V Alloy in NaCl Solution. Corros. Sci. 2016, 102, 484–489. 10.1016/j.corsci.2015.10.041.

[ref49] Sugahara T.; Reis D. A. P.; de Moura Neto C.; Barboza M. J. R.; Perez E. A. C.; Neto F. P.; Hirschmann A. C. O. The Effect of Widmanstätten and Equiaxed Microstructures of Ti-6Al-4V on the Oxidation Rate and Creep Behavior. Mater. Sci. Forum 2010, 636–637, 657–662. 10.4028/www.scientific.net/msf.636-637.657.

[ref50] Seo D.-I.; Lee J.-B. Corrosion Characteristics of Additive-Manufactured Ti-6Al-4V Using Microdroplet Cell and Critical Pitting Temperature Techniques. J. Electrochem. Soc. 2019, 166 (13), C428–C433. 10.1149/2.0571913jes.

